# Co-designing complex interventions with people living with dementia and their supporters

**DOI:** 10.1177/14713012211042466

**Published:** 2021-12-30

**Authors:** Kathryn Lord, Daniel Kelleher, Margaret Ogden, Clare Mason, Penny Rapaport, Alexandra Burton, Monica Leverton, Murna Downs, Helen Souris, Joy Jackson, Iain Lang, Jill Manthorpe, Claudia Cooper

**Affiliations:** Centre for Applied Dementia Studies, 1905 University of Bradford, Bradford, UK; 4784 Alzheimer’s Society Research Network, London, UK; Centre for Applied Dementia Studies, 1905 University of Bradford, Bradford, UK; Division of Psychiatry, 4919 University College London, London, UK; Centre for Applied Dementia Studies, 1905 University of Bradford, Bradford, UK; 53249 NHS England and NHS Improvement London, London, UK; 4784 Alzheimer’s Society Research Network, London, UK; 3286 University of Exeter, Exeter, UK; NIHR Policy Research Unit in Health and Social Care Workforce, 4616 King’s College London, London, UK; Division of Psychiatry, 4919 University College London, London, UK

**Keywords:** co-design, dementia, interventions, public and patient involvement

## Abstract

**Background and objectives:**

We engaged people living with dementia, family carers and health and social care professionals in co-designing two dementia care interventions: for family carers and people living with dementia (New Interventions for Independence in Dementia Study (NIDUS)-family and home-care workers (NIDUS-professional training programme).

**Research design and methods:**

Over October 2019–March 2020, we invited public and patient (PPI) and professional members of our NIDUS co-design groups to complete the PPI Engagement Evaluation Tool (designed to assess engagement activities), and non-professional PPI members to participate in qualitative telephone interviews. We thematically analysed and integrated mixed-methods findings.

**Results:**

Most (15/20; 75%) of the PPI members approached participated. We identified four themes: (1) Creating the right atmosphere: participants found group meetings positive and enabling, though one health professional was unsure how to position themselves within them; (2) Participants influencing the outcome: while most members felt that they had some influence, for one carer consultation seemed too late to influence; (3) Having the right information: several carers wanted greater clarity and more regular updates from researchers; (4) Unique challenges for people living with dementia: memory problems presented challenges in engaging with substantial information, and within a large group.

**Discussion and implications:**

We reflect on the importance of providing accessible, regular updates, managing power imbalances between co-design group members with lived and professional experiences; and ensuring needs and voices of people living with dementia are prioritised. We encourage future studies to incorporate evaluations of co-design processes into study design.

## Background and objectives

Participatory action research is an approach to research that includes the involvement of the community that is being researched in order to understand their world and to ensure that research outcomes are appropriate to identified needs. It describes the principles of participation, cooperation, equality and co-production (Dupuis et al., 2021). In co-production, service professionals, service users and their communities collaborate to improve services and the outcomes people experience. Boviard and Loeffler have described four components of co-production: co-commissioning, co-designing, co-delivering and co-assessing (Robert et al., 2021).

The co-design process has been defined as ‘bringing in the experience of users and their communities to the design of services’. Robert et al. (2021) suggested a wider definition, describing co-design as a ‘complex social intervention whose impacts and outcomes are difficult to evaluate and cannot be reduced solely to the design solutions it generates’, to encompass the potentially transformative effects of co-design.

Co-design has become a ubiquitous term, which may sometimes be co-opted in contexts that sidestep its original principles: distribution of power, amelioration of the human experience and positive societal impact (Moll et al., 2020).These risks are a particular concern when engaging with vulnerable populations. Involving people affected by dementia requires specific consideration – to ensure that family carers and people living with dementia are heard and supported to participate as actively as possible. Dupuis et al. (2016) identified principles and enabling factors necessary to fully support people with dementia in decision-making with others and to help to guide authentic partnerships. These are developing a genuine regard for self and others, recognising the power of synergistic relationships and focussing on the process. Five enabling factors for sustained authentic partnerships emphasise the need for: a diverse group of individuals and identifying and supporting their personal strengths and resources; a safe space where partners feel comfortable expressing their views openly; valuing and including diverse perspectives; establishing and maintaining open communication; and regular critical reflection and dialogue.

Dementia advocacy organisations and individuals contribute considerable resources to supporting research, with the intention of increasing its usefulness to end users. Public and Patient Involvement may include carers, people living with dementia or both, with fewer studies involving the public or professionals (Burton et al., 2019). Several recent publications have illustrated the possibilities and strengths of involving people with lived experience of dementia in co-creation of interventions (O'Connor, 2020), and how participatory action research methods such as the World Café approach, where issues are discussed in small groups, then insights from each group are shared with the larger group, can be helpful in engaging this population (Keogh et al., 2021).

Co-design has been described as a relatively neglected element of co-production that warrants closer attention (Robert et al., 2021). Few studies have formally evaluated the experiences of PPI participants in co-design of dementia care interventions; one previous study recognised the difficulties of power sharing between researchers and family carers of people living with dementia (Rapaport et al., 2018) and another identified practical challenges such as delays with reimbursement and limited access to training resources (Parveen et al., 2018). But there is a dearth of evidence regarding the extent to which authentic partnerships are attained in co-design activities. There is a need to evaluate co-design from the perspective of all stakeholders – not just researchers – if we are to understand how to increase its relevance and utility (Burton et al., 2019).

To our knowledge, this is the first study to investigate the experiences of people living with dementia, family carers and health and social care professionals, working together as co-designers in research. We engaged people living with dementia, family carers and health and social care professionals in the New Interventions for Independence in Dementia Study (NIDUS), and specifically in the co-design of two interventions: (1) for family carers (*NIDUS-family*) and (2) for home-care workers (*NIDUS-professional training programme*). Our interventions aimed to enable people living with dementia to continue living at home as well and for as long as possible, through supporting them, family carers and professionals who help make this possible. In the current study, we aimed to explore the experiences of people affected by dementia of participating in co-design of the NIDUS-interventions.

## Methodology

This study was approved by the University of Bradford (UoB) Ethics Committee (Ref number: E737, 29.01.2020).

### Co-production group recruitment and process

In 2018, we worked with the Alzheimer’s Society Research Network to recruit a NIDUS Community of Interest. This was a forum where stakeholders in the care and support of people living in their own homes with dementia and their family carers could exchange ideas and advise the study, with a remit to ensure its relevance to end users of the research.

Our first annual Community of Interest meeting in April 2018 was attended by 17 family carers of people living with dementia, two home care workers, one Admiral nurse (community nurse specialising in dementia support) and one home care agency manager. Attendees were asked to describe their skills and experience (by completing a form or through a conversation with a researcher) and to indicate how they would like to be involved with NIDUS, with options to participate in co-production through attending project meetings and being a PPI group member, to co-design or to attend Community of Interest meetings only. In collaboration with the Alzheimer’s Society’s PPI coordinator, we then purposively selected, from volunteers, five current or former carers, with diverse skills and experiences, to join our NIDUS-family co-design group (which also included two health professionals and six members of the research team).

#### Co-design processes

**Figure 1.**
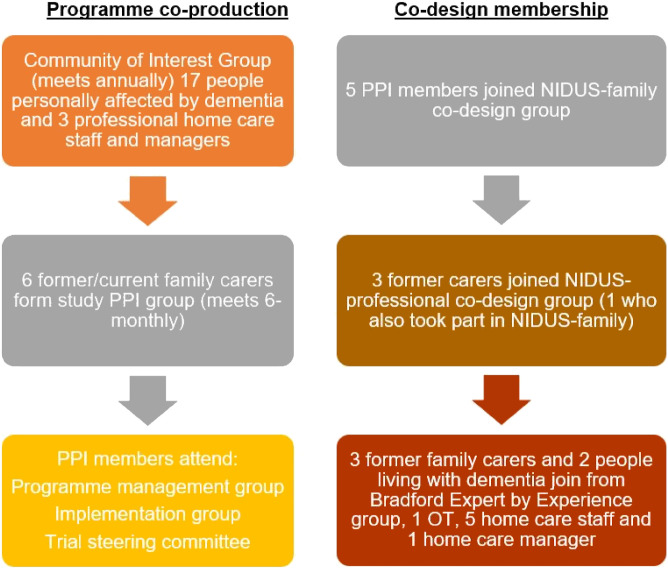
How PPI representatives engaged with the NIDUS programme.

Figure 1 shows how PPI engaged with the NIDUS research programme. Supplementary Table 1 summarises the co-design meetings held, their purpose and outcomes. For **NIDUS-family**, the co-design group met five times, in London: in three workshops to develop the intervention theoretical model, structure and content and two training events to learn together from existing interventions and strategies. The workshops drew on findings from qualitative studies (Herat-Gunaratne et al., 2020; Rapaport et al., 2020a) and systematic reviews undertaken earlier in the NIDUS programme (Lord et al., 2020; Scott et al., 2019), existing interventions (Hassan et al., 2018; Kales et al., 2014; Livingston et al., 2019b, 2019c; Rockwood, 2010) and group members’ expertise. The NIDUS-Family intervention was finalised in March 2020 for delivery in our randomised controlled trial. The final intervention comprises 10 modules, delivered over 6–8 sessions, which cover: (1) Accepting care and Planning for the Future; (2) Communication Strategies; (3) Behaviour & Emotions; (4) Physical Health; (5) Physical Activity; (6) Behavioural Activation and Managing Mood; (7) Carer Well-being; (8) Living Well at Home; (9) Relaxation Techniques and (10) Healthy Sleep and Diet Routines.

We recruited our **NIDUS-professional** co-design group in 2019, from the NIDUS Community of Interest and UoB Experts by Experience group. The NIDUS-professional co-design group initially comprised six former family carers (one of whom also participated in NIDUS-family), two people living with dementia, five home care agency staff, one home care agency manager, an Occupational Therapist and eight members of the research team. The **NIDUS-professional** co-design group met four times: once at Bradford, twice in London and once across both locations. For the first meeting, family carers and people living with dementia met at UoB with half of the research team, and professionals met at University College London (UCL) with the remainder of the research team, and we used video link to enable shared as well as separate discussions. This initial meeting was planned to allow separate discussions and expression of contrasting views between these stakeholder groups, but both groups stated a preference for a shared location at this meeting, so we did not repeat this arrangement. Workshops drew on observational findings from an earlier stage of the NIDUS programme (Leverton, 2020), existing interventions (Kales, 2015; Livingston et al., 2019a; Low et al., 2015; Polacsek et al., 2020) and group members’ expertise. The NIDUS-professional intervention was finalised in September 2020 for delivery in our pilot trial. The final intervention comprises six sessions, which cover: (1) The importance of the home care workers’ role; (2) Building positive relationships; (3) Understanding behaviour as communication; (4) Supporting clients to be as independent as possible; (5) Pleasant activities and teamwork and (6) Bringing it all together and creating action plans.

### Participants

Between October 2019 and March 2020, we invited all members of the NIDUS-professional and NIDUS-family co-design groups who were not from a primarily academic background, to take part in this study. One health professional who co-designed NIDUS-family (HS) is a co-author, and did not complete evaluation questionnaires, while one family carer held roles as a participant in the groups and evaluation as well as a member of the research team (MO). We took these different approaches to enhance the voice of non-professional stakeholders. We thus invited 20 members of the co-design groups to participate: two people living with dementia, six representatives from three home care agencies; one Occupational Therapist, one Nurse and 10 current or former family carers. Participants were offered a £25 voucher following each meeting in appreciation of their time and contribution, and travel expenses were met. We received written, informed consent from all participants, after they had an opportunity to review the study information sheet that was given to all participants. Consent was obtained at the outset of data collection; researchers were trained to review consent at all subsequent data collection points.

### Data collection

In order to reduce risk of bias in this UCL-led study, interviews were conducted by experienced qualitative researchers based at UoB (DK, KL, CM). All participants were sent a study information sheet either via email or post and given 72 hours to decide if they wished to participate. Socio-demographic characteristics were recorded; then participants completed the Public and Patient Engagement Evaluation Tool (PPEET), version 2 (August 2018) (Abelson et al., 2016). The authors removed tool elements irrelevant to participants’ role in NIDUS co-design. PPEET comprises Likert-style (Supplementary Table 2) and open-ended questions about how their participation in the co-design group was supported, whether they were able to share their views, the influence of the co-design group and their role within it on the project and what could be improved about the co-design process.

In addition to questionnaire completion, we also invited participants who were family carers or people living with dementia to take part in semi-structured telephone interviews, using a topic guide. The topic guide was developed by the authors based on the literature and included items identified in Guidance for Reporting of Public and Patient Involvement (GRIPP) criteria (Abelson et al., 2016). The topic guide was piloted with a PPI representative (MO).

### Analysis

We summarised responses to discrete choice PEET questions as frequencies. As numbers were small, we did not conduct quantitative analyses. Free responses to PEET questions are fully reported (Supplementary Table 3, Appendix). We digitally audio-recorded qualitative interviews and transcribed them verbatim, removing identifying information from transcripts prior to analysis. We used the qualitative research software NVivo 12 to code, manage and analyse data. Four researchers, one of whom was a PPI representative (KL, MD, MO and CM), thematically coded all data independently to ensure reliability, generating a coding frame from initial interviews using a thematic content analytic approach. Discrepancies between the researchers were resolved through discussion and a consensus was reached. KL and CC then used methods similar to those described by Moran-Ellis et al. (2006), termed ‘Following a Thread’, to integrate data from the questionnaire (quantitative and qualitative) within this framework.

## Results

Fifteen of the 20 co-designers approached participated in either the questionnaire or qualitative telephone interview. The 10 family carers were all from a White ethnic background; one was male and nine were female; they were aged between 52 and 84 years. Six completed the questionnaire and interview study components; two only completed the questionnaire and two only the interview. We interviewed one man and one woman living with dementia, of whom one completed the questionnaire with support from CM and both took part in interviews; one was aged 61 years and the other preferred not to say. Interviews were between 20 and 50 minutes in duration. Participants were offered the opportunity to review their interview transcripts; however, no participants did. Three professionals took part: an Occupational Therapist, a home care agency manager and home care worker (from two different agencies). Two professionals were female, and one was male; they were aged between 40 and 57 years. All five potential participants declining participation were professionals (four agency staff, one nurse). Of those who declined, one did not attend any sessions (though commented on some materials) and three home care agency professionals only attended the first session.

Responses to Likert questionnaire items are displayed in Supplementary Table 2 and free-text responses are listed in Supplementary Table 3 (Appendix). We present below our findings, integrated across questionnaire responses and qualitative interviews.

### Findings of thematic analysis

We identified four over-arching themes in the data: Theme 1: Creating the right atmosphere; Theme 2; Feeling that group members influenced the outcome; Theme 3: Having the right information and Theme 4: Unique challenges for people living with dementia in the co-design process.

### Theme 1: Creating the right atmosphere

All participants described how facilitators created the right atmosphere and fostered positive group dynamics. They spoke of the warm, receptive, down-to-earth nature of the group:

‘The people running the project are very approachable and very welcoming and they are really nice and easy to work with’ [Family carer, ID06, interview]

‘The professionals are a caring group of people who are good listeners. They are a very friendly group of people who have a lot of respect for each other & obviously get on very well with each other’. (Family carer, ID10, questionnaire)

Participants also appreciated the approach of the research team who provided reassurance, made them feel comfortable and valued and enabled them to express their views and opinions:

‘The researchers made us feel very comfortable and we were in a safe place and we could talk freely’ [Family carer, ID04, interview]

‘You can say anything – it’s just a case of saying it. No one judges you. You can say what you feel’ [Person living with dementia, ID11, interview]

This atmosphere sometimes led to participants at the workshops feeling able to disclose very personal experiences that led to perceived benefits for that individual:

‘I had kind of forgotten all about it but it was good to talk about it… because for a lot of carers you don’t get the opportunity because your friends and relatives don’t want to know and so it is probably, yea, it is a really selfish thing but actually doing this has been a really cathartic thing to me personally and it’s been great’ [Family carer, ID01, interview].

By contrast, one of the professionals involved in the NIDUS-professional workshops commented that it was hard to know how to position themselves within the group:

‘New experience for me - wasn’t quite sure at times re how to position myself amongst the other co-production participants - perhaps could have met individually with researchers initially?’ (Professional, ID12, questionnaire)

Some participants reflected on the iterative nature of research and the co-design process highlighting that it can often be a gradual ‘learning curve’ for participants, members and researchers alike:

‘I think it was a bit of a learning curve, if I’m honest, for both the researchers and for the PPI because I think neither one of us knew really what were going to be our roles’ [Family carer, ID04, interview]

While questionnaire responses indicated that most group members felt they heard a wide range of views within the group, one noted a lack of diversity:

‘Mostly older people & white British’ (Family carer, ID07, questionnaire)

In summary, most participants experienced the co-design groups as welcoming and facilitative, though the health professional interviewed was less sure of her position within them. A lack of ethnic diversity within the group was acknowledged.

### Theme 2: Feeling that group members influenced the outcome

Questionnaire responses indicated that most participants felt the co-design groups had impact and that their work made a difference (Supplementary Table 2). Impacts cited included conveying their lived experience and influencing the intervention manual content:

‘As a family carer I was able to bring experience of caring for my mother who had dementia and I felt some things were changed as a result of being listened to’ (Family carer, ID04, questionnaire)

‘Comments on manual taken on board’ (Family carer, ID05, questionnaire)

‘Put forward what I would want to see - if I had a carer (care worker) what would I want. And what wouldn’t I want’. (Person living with dementia, ID11, questionnaire)

‘I feel I have given valuable insight into the project and home care’ (Professional, ID14, questionnaire)

Most participants felt that they were there to genuinely influence the discussions and that their views were taken on-board by the research team:

‘You know, they are a nice team to work with, everybody was. So we weren’t just there as token…it wasn’t tokenistic or anything like that. No, I think we did bring our views and I think they were taken on-board and I think we all…I think that was one of those where we worked quite well’ [Family carer, ID04, interview]

Two NIDUS-family participants said that they would have liked more information about the impact their involvement had made:

‘I would have liked to be kept informed of the progress after my involvement ended’. (Family carer, ID04, questionnaire)

*‘On that basis… I’m pretty ignorant of the effect I’ve had’* (Family carer, ID03, interview]

In summary, participants felt their contributions were heard and considered, and while most were confident that they would be reflected in the intervention design, others were less sure and would have liked more communication after the design process had ended about how their ideas would be used.

### Theme 3: Having the right information

Co-design workshops were held several weeks apart and contact in between with participants varied. While most respondents indicated that they understood the purpose of NIDUS and had sufficient support and information, three family carers who participated in NIDUS-family co-design indicated that they would have liked more communication, before and between meetings:

‘Periods of silence in between meetings were too long… hard for non-academics to keep track of where we were [with the project]’ [Family carer, ID05, questionnaire)

‘I didn’t really know what was happening… You still feel some involvement in it, but it’s, sort of, been…I feel, as you say, a bit distanced from it’ (Family carer, ID04, interview)

One of these family carers felt PPI members had less clarity than researchers about what was happening, and this could be a barrier to meaningful engagement:

‘I think sometimes the research, you know, it was slightly muddled…sometimes get a bit confused about how the manual was supposed to work, at what…which point it was going to be used when… You know what I mean? Not necessarily something that we would prepare for because it was all totally clear to them. I suppose that’s partly the process…’ [Family carer, ID05, interview]

Another family carer echoed these calls for clarity around opportunities to feedback as well as information about how existing interventions might inform the NIDUS-professional manual:

‘More notice of dates of meetings if possible. At Nov(ember) meeting, I had prepared some comments on Session 1, but there did not seem to be an opportunity to provide this feedback. Since materials from (a previous study) are being used in the training programme, I’d like to know more about that’. [Family carer, ID03, questionnaire]

More consistent contact between meetings and regular project updates were suggested as improvements. As NIDUS is a large programme of work, participants requested a visual timeline of progress of the whole project so that they had a better sense of what was happening in all streams of the programme:

‘I think possibly it would be helpful just to have, like, a process chart, something that maybe maps out where you are in an update, even a tiny little diagram. I don’t mean something laden with words or whatever, but ‘this is where we are an this is where we hope to get by…’… a visual thing’ [Family carer, ID05, interview].

One family carer felt that decisions around the content of the interventions had already been made by researchers before they were consulted:

‘I got the impression that I was telling them probably useful information but too late. I think they’d already made up their mind what they wanted to do and it was too late to take on-board any further even if it might have been useful’ [Family carer, ID03, interview]

In summary, several participants wanted more information. The research and design processes sometimes felt confusing, and difficult to navigate. A visual map of the process was suggested.

#### Theme 4: Unique challenges for people living with dementia in co-design process

All participants valued the presence and contribution of the two people living with dementia and appreciated the insights from hearing the differences in their views and experiences:

‘We actually had a couple of people who actually had dementia which was fantastic. I think it probably helped us because the rest of us were carers… they had a totally different perspective’ [Family carer, ID01, interview]

‘Hearing from people with dementia was particularly powerful. Knowing what things are important to them was really good’ [Family carer, ID07, interview]

However, people living with dementia highlighted some of the challenges of being asked to contribute to large group discussions given their ability to retain information:

‘One thing that really gets me, that upsets me, is having no memory. Or not having enough memory to remember what I need to remember, it’s so frustrating. You think you’ve got the information in your head and when you want to say it, it’s gone’ [Person living with dementia, ID15, interview]

People living with dementia also noted challenges with others in the group either being quicker to speak or speaking over them on occasion:

‘The biggest problem I have is something will come into my head, but when I go to speak, someone will speak over me, or start talking before me, and then I lose my train of thought and then when I’m asked do you want to say something, well its gone, and I can’t remember what I was going to say’ [Person living with dementia, ID11, interview]

One person living with dementia felt that the workshops were a little long at times, especially if they felt that they did not have anything to contribute to a specific topic:

‘Meetings can be very long and sometimes drifted into other discussions which went over my head and weren’t for me to contribute to. That would be okay as long as they said that – then you would feel like you could rest for a minute or two instead of making lots of effort to try and follow everything when it’s not actually relevant to you’ [Person living with dementia, ID11, interview].

In summary, for people living with dementia, workshops sometimes felt long; they would have found it helpful for their contributions to be prioritised, with less time spent in sessions to which they did not feel able to contribute.

## Discussion

We sought to develop inclusive co-design processes that enabled all participants to engage meaningfully in the design process. Our findings indicate we partially achieved this goal, but that two groups felt less supported to engage meaningfully: health professionals and participants living with dementia.

Health professional described feeling unsure about how to position themselves. This is to our knowledge the first formal evaluation of co-design involving professionals, family carers and people living with dementia. While most of the family carers recruited had previous experience of PPI work, the professionals and home care staff did not. The home care workers interviewed did not mirror the health professional’s concerns, but as four declined participation, they may have shared this discomfort.

Family carers spoke in NIDUS-professional workshops about negative previous relationships with home care services, and this could have been uncomfortable for home care workers. This potential issue contributed to our decision to hold the first NIDUS-professional workshop with these groups separately located but video-linked. Co-design groups were one component of PPI engagement in the NIDUS programme, in which we co-created values, co-designed interventions and co-produced research. Williams et al. (2020) has modelled the inter-relationships of these processes in a healthcare context. As most co-designers who were experts by academic or lived experience were already involved in the NIDUS programme as PPI members, the professional co-designers may have felt positioned as relative outsiders. As suggested by the health professional participant, initial individual meetings between co-designers and the research team would have been beneficial, so those new to co-design knew what to expect.

**Figure 2.**
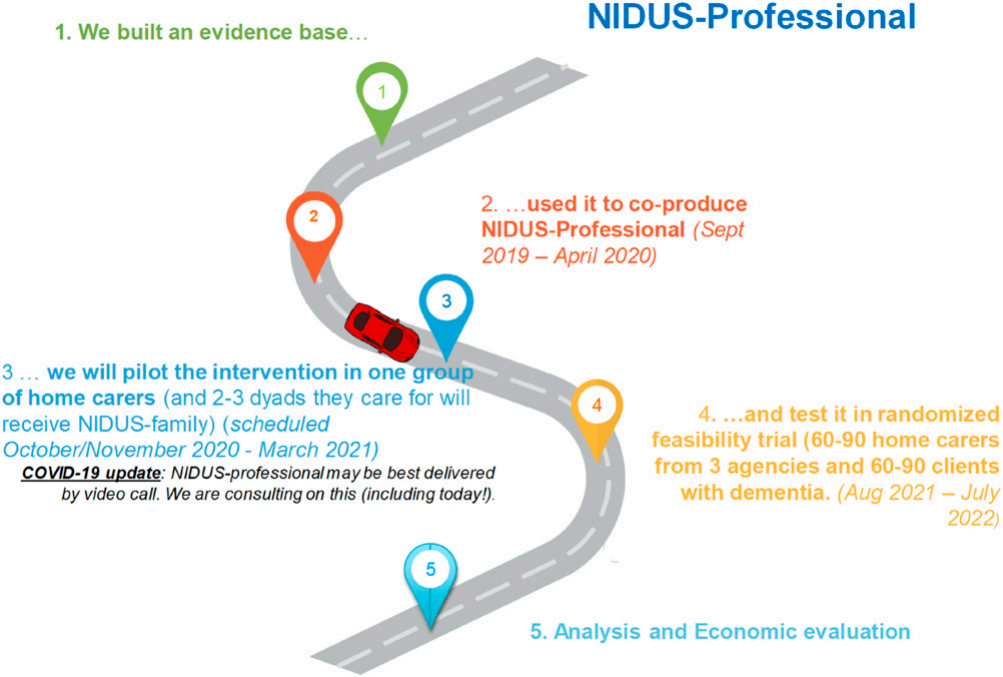
Example of new visual project presentation introduced in response to PPI feedback.

The reports from co-designers who lived with dementia highlighted barriers to participation that memory difficulties present and suggested how they may be reduced: by prioritising contributions of people living with dementia and ensuring that information is presented clearly and simultaneously. We sought to manage information overload around meetings (CM visited participants living with dementia to work through documentation with them) but in workshops they sometimes found it challenging to interject and be heard. We have taken up one suggestion of the group to present information in a more visual format, as a roadmap at PPI meetings (example in Figure 2). Several participants from varied backgrounds would have liked more, and more regular information. An explicit conversation at the start of co-design about what information each co-designer would like and how frequently, would have helped ensure they received a sufficient, but not overly burdensome, amount of information. While we strove to develop authentic partnerships with participants (Dupuis et al., 2016), these findings suggest room for improvement; we discussed how we would work together as a group at the outset of the design processes, but on reflection will supplement this with an individual approach in future projects.

Balancing the needs of a large research project with pre-determined ideas about interventions and tight timelines with the creative and time-consuming nature of co-design can be challenging. The co-design process was research team-led, and professionally dominated (Ocloo & Matthews, 2016), although we sought to reduce this power imbalance. We explicitly acknowledged different areas of expertise. For example, PPI members’ views and knowledge about intervention acceptability were prioritised. Where members suggested additional material or topics, this was explicitly balanced against the requirement to design a realistically deliverable intervention. Academic and clinician expertise on feasibility was also acknowledged. We tried to be explicit about decision-making, but our findings indicate we did not always achieve this. We sought to avoid positioning academics as sole guardians of the ‘evidence-base’, through engaging the group in planning how findings from research conducted to inform workshops would be used. For example, observation study findings were presented alongside fieldnotes and transcript excerpts, from which members selected material to include in the intervention – as case studies or quotes (Leverton et al., 2019). The group also trained together on interventions that could inform the NIDUS-interventions (Supplementary Table 1).

Prior to commencement of the main NIDUS-family trial and the NIDUS-professional pilot trial, the COVID-19 pandemic necessitated a move to remote online delivery. We consulted our Community of Interest and PPI group about how to do this in April/May 2020, after data collected for the current study was complete. These discussions were critical to the success of our move to remote delivery, and highlighted the important ongoing role of PPI members in research, beyond the co-design presented here. PPI members were also subsequently involved in recruiting and training facilitators for the pilot and main intervention trials. We describe the content of the final NIDUS-family intervention elsewhere (Rapaport et al., 2020b). A definitive RCT is currently underway (Burton, 2020).

## Strengths and limitations

A key strength of this evaluation is the involvement of people affected by dementia in its conception, analysis, interpretation and write-up. Family carers participating included those who were sole carers, shared caring with another family member and those caring alongside professionals and care workers. Although participants said they felt comfortable voicing their opinions, a limitation of this work is that researchers who were interviewers also participated in the co-design workshops.

Limitations of the co-design process, which this paper evaluates, included the failure to recruit people living with dementia to the NIDUS-family co-design group. Relatively few of the professionals who attended workshops participated, so we can be less confident that the views expressed by this group are representative of professionals involved in this co-design. This study has evaluated the co-design process. While we explain how the wider co-production of the NIDUS programme, which is ongoing, interfaced with co-design, we did not evaluate it. The challenges we discuss within co-design may have relevance for the wider programme, for example, in the delivery and governance of the final product, and we will seek to learn from them. Our final caveat regards use of the term ‘co-designer’. We found that the involvement and sense of influence of participants varied. This term suggests a level of shared decision-making, power and responsibility that may for some PPI participants, exceed the realities of their experiences.

## Conclusions

We have co-designed two complex interventions with people affected by dementia. In this study, we sought to learn from the process. PPI members provided useful feedback about how we could adapt our processes to facilitate their engagement: specifically, through more information provision. Another area of learning was the importance of managing power imbalances between PPI groups and members with lived and professional experiences. We encourage more projects developing interventions to evaluate their process of co-design throughout in order to adapt and develop their approach as the work progresses.

## Supplemental Material

sj-pdf-1-dem-10.1177_14713012211042466 – Supplemental Material for Co-designing complex interventions with people living with dementia and their supporters Supplemental Material, sj-pdf-1-dem-10.1177_14713012211042466 for Co-designing complex interventions with people living with dementia and their supporters by Kathryn Lord and Daniel Kelleher, Margaret Ogden, Clare Mason, Penny Rapaport, Alexandra Burton and Monica Leverton, Murna Downs, Helen Souris, Joy Jackson, Iain Lang, Jill Manthorpe in Dementia
