# Supplementary material for: Co-designing complex interventions with people living with dementia and their supporters
Source: Dementia (London). 2021 Dec 30;21(2):426–41. doi: 10.1177/14713012211042466 (PMC8811333; doi:10.1177/14713012211042466)
Supplement: sj-pdf-1-dem-10.1177_14713012211042466 – Supplemental Material for Co-designing complex interventions with people living with dementia and their supporters [file sj-pdf-1-dem-10.1177_14713012211042466.pdf]

Supplemental data: Table 1: Co-design workshops and training events held

Date	Purpose	Outcomes
**NIDUS-family**		
12.9.18	Present systematic review and research findings (Herat-Gunaratne et al., 2020, Rapaport et al., 2020a, Scott et al., 2019, Lord et al., 2020); share draft theoretical model and seek PPI input<br>Agree plan to develop intervention and content	PPI members requested additional searches to develop theoretical model (Lord et al., 2020)<br>Intervention structure agreed<br>PPI members asked researchers to draft a plan for intervention content that they could comment on "don't give us a blank piece of paper"
30.10.18	Training by Ken Rockwood on Goal Attainment Scaling (GAS): The primary outcome of the NIDUS-Family trial (Rockwood, 2010)	Developed understanding of how intervention needs to fit with participants' goals
31.10.18	Training by Helen Kales and team on DICE approach (Kales et al., 2014)	Understanding of how we could incorporate DICE as a key component in intervention
26.11.18	Research team share plan for intervention structure and content	PPI gave feedback on plans; group agreed research team would develop a first draft of whole intervention to share at next workshop
30.1.19	Research team share first draft of intervention	PPI gave detailed comments to research team to inform next draft, and agreed to iterate further by email/ phone discussions
**NIDUS-professional**		
23.9.19	Discussion of knowledge, skills and support home carers need; and how we will work together; learning from Promote Independence Through quality dementia Care at Home (PITCH) team materials (Polacsek et al., 2020)	Agreed top-level plan for content of 6 session intervention<br>Research team to draft session 1 and circulate to group
28.11.19	Discussed session 1 draft and content to prioritise in next sessions<br>Small group discussions about possible content to include in sessions 2-4; Discussed how we can learn from NIDUS-family	Research team to draft sessions 2-4 and circulate to group ahead of next meeting<br>Research team to review session 1 as per group feedback
22.1.20	Discussed sessions 2-4 drafts and content to prioritise in next sessions<br>Discussed learning from University of Bradford training materials	Research team to draft sessions 5-6 and circulate whole intervention to group ahead of next meeting
11.3.20	Discussed the draft intervention: what needs to feed and how to feedback; discussed content of facilitator training and process of engaging home care agencies<br>Discussed how we can learn from the lifestyle engagement activity program (LEAP) materials (Low et al., 2015)	Research team to finalise intervention manual; Develop a guide based on discussion for training and agency engagement; agreed for group to meet again to review results of intervention pilot (in 2021)

Supplemental data: Table 2: Adapted Public and Patient Engagement Evaluation Tool questions, Likert scale responses

**Questions**	**Strongly agree**	**Agree**	**Neither agree/ disagree**	**Disagree**	**Strongly disagree**
I have a clear understanding of the purpose of NIDUS	7	6	0	0	0
The supports I need to participate in NIDUS are available.	8	4	1	0	0
I have enough information to be able to carry out my role.	5	7	1	0	0
I am able to express my views freely.	9	4	0	0	0
I feel that my views are heard.	9	4	0	0	0
A wide range of views on discussion topics is shared.	6	7	0	0	0
The individuals participating in NIDUS represent a broad range of perspectives.	6	6	1	0	0
This co-production group is achieving its stated objectives.	2	8	3	0	0
I am confident the research team takes the feedback provided by the group into consideration.	9	3	0	1	0
I think the work of the co-production group makes a difference to the NIDUS project.	7	6	0	0	0
As a result of my participation, I am better informed about the needs of people living with dementia.	4	6	3	0	0
Overall I am satisfied with this engagement initiative.	4	8	1	0	0
This engagement initiative is a good use of my time.	6	5	1	1	0

Supplemental data: Table 3: Adapted Public and Patient Engagement Evaluation Tool (PPEET) questions NF = NIDUS-family; NP = NIDUS-professional

**What else would you like us to know about how your participation in the co-production group was supported?**
I would have liked feedback from the meetings and an agenda for the following meeting well in advance (family carer, NF)
Academic team should provide more information in between sessions (family carer, NF)
I felt that I would be able to ask any questions if I needed support to part in the NP co-production. Questions we answered clearly. Thank you. (family carer)
More info re [another intervention] - what materials have come from this project? (family carer, NP)
Plenty of information. Need to sure we keep it relevant. End of session started talking about management and that went over my head. I lost interest (person living with dementia, NP)
New experience for me - wasn't quite sure at times re how to position myself amongst the other co-production participants - perhaps could have met individually with researchers initially? (professional, NP)
It has been very interesting and exciting to be part of (home care worker, NP)
**What else would you like us to know about how you are able to share your views?**
I felt it was a safe environment to discuss our views (family carer, NF)
I am not always confident that I have much to contribute (family carer, NP)
I feel that my views were listened to and valued. (family carer, NP)
Mostly older people & white British (family carer, NP)
Adapting to various difficulties as they arise (family carer, NP)
It's a case of just saying it. No one judges you. You can say what you feel. (person living with dementia, NP)
Possibly 1-1 initially would have been helpful (professional, NP)
**In your role, what influence do you think you have had to date?**
I've provided general support to the research team (family carer, NF)
As a family carer I was able to bring experience of caring for my mother who had dementia and I felt some things were changed as a result of being listened to (family carer, NF)
Comments on manual taken on board (family carer, NF)
A small influence. Possibly regarding the quality of the training and calibre of outside carer (family carer, NP)
Considering the sort of carer I would like to care for my husband and sharing with the group (family carer, NP)
My experience as a carer with home care (family carer, NP)
Experience of living with someone with dementia (family carer, NP)
I feel as in my role as a carer I have been able to pass on some information about the day to day problems of living with someone with dementia (family carer, NP)
Put forward what I would want to see - if I had a carer what would I want. And what wouldn't I want. (person living with dementia, NP)
Made suggestions of using video for training?? (professional, NP)

I have been able to share my experiences (professional, NP)<br>I feel I have given valuable insight into the project and home care (professional, NP)
**What are the strengths of the co-production group?**<br>Groups have been big enough to a variety of views but not too big to make you feel insignificant (family carer, NP/F)<br>That it takes into account the experience of a cross section of family carers (family carer, NF)<br>Able to speak freely about concerns, being an influence on my role as a family carer. Making me more aware of the person with dementia & their independence (family carer, NF)<br>Cross section of participants (family carer, NP)<br>Diversity of contributors - all interested parties involved. The professionals are good listeners and value the opinions of others. (family carer, NP)<br>Involving people with dementia and different kinds of dementia is excellent (family carer, NP)<br>Variety of roles & experiences from various working backgrounds (family carer, NP)<br>The professionals are a caring group of people who are good listeners. They are a very friendly group of people who have a lot of respect for each other & obviously get on very well with each other. (family carer, NP)<br>Felt listened to and given time to put point across (person living with dementia, NP)<br>Input from all concerned parties (professional, NP)<br>The whole group participation (professional, NP)<br>Open to suggestions and eager to learn and improve training (professional, NP)
**What could be improved about the co-production workshops?**<br>To focus on the early training (at the time of early signs of dementia - preferably pre diagnosis) of the prime carer to ensure they understand the disease, how to look after themselves and personal centred care of the person with dementia. (family carer, NF)<br>Difficult as we all took different things from it I suspect but found it very helpful. I would have liked to be kept informed of the progress after my involvement ended. (family carer, NF)<br>More communication in between workshops. Stronger sense of what changes have been made as a result of groups thoughts, comments, suggestions. Periods of silence too long - hard for non-academics to keep track of where we were (family carer, NF)<br>I was happy with the workshop especially when the whole group worked face to face. (family carer, NP)<br>More notice of dates of meetings if possible. At Nov meeting, I had prepared some comments on Session 1, but there did not seem to be an opportunity to provide this feedback. Since materials from MARQUE are being used in the training programme, I'd like to know more about that. (family carer, NP)<br>Nothing - we were listened to. With a lovely bunch of knowledgeable people. Easy to take in and understand. (family carer, NP)<br>I don't think it could be improved, it worked very well especially meeting face to face at the meetings in London & Bradford. (family carer, NP)<br>Keeping it relevant and not drifting into other discussions. It's okay if you set it up and give the option of listening; "this may not be relevant to all of you, but" - I'd know I could rest for a minute and focus when it's more important for me to contribute (person living with dementia, NP)
